# Tumor Burden and Immunotherapy: Impact on Immune Infiltration and Therapeutic Outcomes

**DOI:** 10.3389/fimmu.2020.629722

**Published:** 2021-02-01

**Authors:** Samuel I. Kim, Christopher R. Cassella, Katelyn T. Byrne

**Affiliations:** ^1^ Program in Biochemistry, College of Arts and Sciences, University of Pennsylvania, Philadelphia, PA, United States; ^2^ Department of Medicine, Perelman School of Medicine, University of Pennsylvania, Philadelphia, PA, United States; ^3^ Parker Institute for Cancer Immunotherapy, University of Pennsylvania, Philadelphia, PA, United States

**Keywords:** tumor burden, immunotherapy, immune infiltrate, tumor microenvionment, immunosuppression

## Abstract

Cancer immunotherapy has revolutionized the treatment landscape in medical oncology, but its efficacy has been variable across patients. Biomarkers to predict such differential response to immunotherapy include cytotoxic T lymphocyte infiltration, tumor mutational burden, and microsatellite instability. A growing number of studies also suggest that baseline tumor burden, or tumor size, predicts response to immunotherapy. In this review, we discuss the changes in immune profile and therapeutic responses that occur with increasing tumor size. We also overview therapeutic approaches to reduce tumor burden and favorably modulate the immune microenvironment of larger tumors.

## Introduction

Nearly 50 years ago, Whitney and colleagues reported that mice bearing large carcinogen-induced sarcomas displayed defective spontaneous immune responses (1). These immunological defects were reversible with tumor resection, providing a link between tumor burden and dysfunctional immune responses. In the intervening decades, further interrogation into the immunobiology of the tumor microenvironment (TME) reveals significant local and systemic impacts on immune responses (2, 3). With the advent and success of immune checkpoint blockade (ICB) in patients with advanced disease (4), there is a need to understand biomarkers of response to improve outcomes in patients that remain resistant to immunotherapy. Despite the identification of several correlates of response to ICB, including CD8 T cell infiltration of the tumor site, tumor mutational burden, and cytolytic T cell gene expression profiles (5–7), these metrics are not universal predictors of response across all tumor types and patient subsets. Here, we review the literature with regard to total tumor burden as an important negative correlate of response and explore potential mechanisms to mitigate the local and systemic impact of high tumor burden on immune interventions.

## Tumor Burden Impacts Baseline Immunity

The complex immunobiology of the TME is regulated by a number of factors, including tumor cell-intrinsic determinants of immune cell infiltration (8), the tissue in which the tumor is located (9), and the stromal and vasculature content of the tumor [reviewed elsewhere (10, 11)]. Immune cell intrinsic defects also contribute to tumor progression, including T cell exhaustion or tolerization to high avidity tumor antigens (12). This multitude of factors contribute to tumor progression, but preclude mechanistic insight in heterogeneous clinical samples, even across tumors harvested from a single patient (13). Mouse models provide crucial insight into the impact of tumor burden on immune features in the TME by allowing for isolation of the impact of tumor size as a variable regulating immune infiltration and immunotherapy sensitivity. Preclinical data support the clinical findings that large tumors are more immunosuppressive compared to small tumors on both the local and systemic level, directly impacting the ability of the host immune system to effectively mount natural or immunotherapy-induced immune responses.

The immunosuppressive nature of the tumor site is locally enforced *via* immune cell subsets recruited or induced within the TME. Pro-tumorigenic myeloid derived suppressor cells (MDSCs) mediate T cell suppression through a variety of mechanisms including depletion of arginine, oxidative stress of target cells, and release of the dicarbonyl radical methylglyoxal (14–16). MDSCs have been found to increasingly infiltrate the TME in a murine model of renal adenocarcinoma (RENCA) even as the populations of other immune cells (including T cells and dendritic cells; DCs) decreased (17, 18). Similarly, in a murine model of pancreatic ductal adenocarcinoma (PDAC), MDSCs and tumor-associated macrophages have been shown to increasingly infiltrate the TME as tumors progress (19). The same trends are observed in regulatory T cells (Tregs) in multiple tumor types, concurrent with a decrease or even absence of CD8 T cells, NK cells, and DCs during tumor progression (17, 19–22). These data suggest that targeting suppressive immune cell populations may help convert the TME of large tumors to more closely resemble the immune infiltrate of small tumors, augmenting the impact of immunotherapeutic interventions.

In addition to increased proportions of immunosuppressive cells in the TMEs, the cytokine production in large tumors is also skewed to a more suppressive profile as compared to small tumors. Transforming growth factor-β (TGF-β), a pleiotropic cytokine shown to exert anti-tumor effects in early-stage cancer but tumor-promoting effects in late-stage cancer (23), is amplified in large murine T cell leukemia and RENCA tumors (17, 24). IL-10 and nitric oxide synthase 2 (NOS2) similarly increase as tumors progress (17, 24). This likely reflects progressive increases of MDSCs and Tregs in growing tumors (19–22), as both cell populations are major producers of these suppressive cytokines.

However, not all TMEs follow the same positive correlation between suppressor cell infiltration and increased tumor size. In patients with colorectal cancer, CXCL9 – an IFN-γ inducible chemokine that recruits CD8 T cells – is not differentially expressed according to high or low tumor burden (25). In a mouse model of melanoma, B16 tumors show very little perturbation in the relative frequencies of effector or regulatory T cell subsets as tumors increase in size (17). Similarly, CD8 T cells are actually increased (while Tregs are decreased) in large vs. small tumors in a mouse model of colon carcinoma (17). However, chronic antigen exposure has the potential for tumor-specific T cell deletion, resulting in functional “holes” in the immune cell repertoire, a mechanism first reported in the context of chronic viral infections (26). High avidity T cell interactions also have the potential for deletion, as observed in a mouse model of lymphoma where tumor-specific T cells were rapidly lost in the context of a highly immunogenic tumor antigen when the number of target tumor cells was above a certain threshold (27). The failure of these effector cells in large tumors highlights the contribution of other suppressive mechanisms – beyond those mediated by sheer numbers of suppressive MDSC or Treg populations – in established and progressing tumors.

One such mechanism by which tumors escape T cell-mediated destruction is *via* negative immune checkpoints including programmed death-1 (PD-1) or cytotoxic T lymphocyte-associated protein 4 (CTLA-4). Ligation of immune checkpoints on T cells drives exhaustion and dysfunction, preventing autoimmunity and immunopathology in the context of extenuating immune activation, but usurped by the tumor to disable anti-tumor immunity. Immune checkpoint ligands such as programmed death ligand 1 (PD-L1) are abundant in the TME of many tumor types, independently of tumor mutation burden (28). Multiple tumor types exhibit increased PD-L1 expression as a direct correlate with tumor size including gastroenteropancreatic neuroendocrine tumors (29), advanced gastric cancer (30), and meningiomas (31). Furthermore, in primary and metastatic melanoma samples, PD-L1 levels are higher in primary and local metastases and lower in distant metastases (32) with similar findings in cutaneous squamous cell carcinoma (33) suggesting that expression of checkpoint ligands may be linked to tumor progression.

The immunosuppressive impacts of bearing a large tumor are often not limited to the local TME. Recently, Allen and colleagues revealed that the presence of a tumor negatively impacts the systemic immune landscape, including a global suppression of T cell responses to tumor-unrelated antigen challenges as a result of increased IL-1 and G-CSF (3). Similarly, conventional dendritic cells responsible for priming CD8+ T cells were found to be systematically and progressively dysregulated in mice bearing PDAC, leading to deficient T cell priming even in the presence of strong tumor neoantigens (34, 35). In murine PDAC, tumor cell-derived G-CSF impaired differentiation of type 1 conventional dendritic cells in the bone marrow and blood (36), and elevated serum levels of the cytokine IL-6 in tumor-bearing mice induced apoptosis of conventional dendritic cells (34). Thus the findings of failed systemic immunity in mice bearing large tumors have come full circle (1), revealing that both local and global suppression of the immune response is heightened in the context of increased tumor burden. Targeting both deficits may provide the signal necessary to drive immune responses in currently treatment-resistant tumor types.

## Impact of Tumor Burden on Therapy-Induced Immune Responses

Spontaneous or natural cancer-immunity cycles have failed by the time a tumor is detected in the clinical setting, requiring therapeutic interventions to drive immune-mediated rejection of tumors. ICB has thus far garnered the most success and has shown remarkable clinical benefit in patients with aggressive disease (4), but gene therapy approaches such as chimeric antigen receptor (CAR) T cells have become standard-of-care for some patients with previously incurable malignancies (37), and oncolytic viruses have shown great promise in treatment-refractory brain tumors (38). Integrating knowledge about the impact of tumor burden on the efficacy of these therapies may enhance clinical outcomes.

### Immune Checkpoint Blockade

Immune checkpoints such as PD-1/PD-L1 or CTLA-4 are negative regulators of activated T cells. Using monoclonal antibodies (mAbs) to disrupt the ligand/receptor pairing of these immune checkpoint molecules enables tumor-associated T cells to overcome immunosuppression and effectively perform anti-tumor functions (39). ICB has drastically improved clinical outcomes for patients with advanced disease including metastatic melanoma (40–42) and non-small cell lung cancer (NSCLC) (43–45).

Despite the immense positive impact of ICB in the clinical setting, many patients do not respond, and tumor burden is one metric that negatively correlates with ICB efficacy. Preclinical data reveals that PD-1 blockade is more effective in mice bearing smaller lung squamous cell tumors (22). Similarly, mice with advanced ovarian tumors are more resistant to PD-L1 blockade than mice with earlier stage tumors (21, 46). This negative correlation of tumor size and ICB sensitivity is borne out in patients where total tumor volume is predictive of response to αPD-1 with local or metastatic melanoma (47, 48). In patients with NSCLC, metabolic tumor volume was also a prognostic factor for sensitivity to PD-1 blockade in both retrospective and prospective studies (22, 49). Furthermore, dual ICB, which targets both PD-1 and CTLA-4 at the same time, has shown greater efficacy than single ICB in patients with metastatic NSCLC and melanoma (40, 43), and is more effective in melanoma patients with smaller baseline tumor diameters (50). Despite the plethora of studies correlating tumor size and ICB outcomes, the threshold of tumor burden at which ICB efficacy is reduced has not been defined. Given this, a dynamic metric, such as the ratio of proliferating T cells specifically reinvigorated by anti-PD-1 compared to the total tumor burden (51) may be the best approach for identifying patients that are resistant to ICB and would benefit from additional therapeutic interventions.

### T Cell Costimulation

In contrast to alleviating suppressive signals *via* ICB, direct co-stimulation of T cells using mAbs targeting molecules such as OX40 and 4-1BB has also been explored. OX40 is a member of the TNF receptor superfamily expressed by activated T cells, and agonistic OX40 mAbs have direct stimulatory activities on effector T cells with the benefit of inhibiting Treg function in the tumor site, showing early promise in clinical trials (52). Preclinical studies reveal that small MCA205 fibrosarcoma tumors and CT26 colon carcinomas are sensitive to OX40 agonism as a single agent, but larger tumors (50–120 mm^2^) require the addition of transforming growth factor-β (TGF-β) receptor antagonists to further reduce immune suppression (53). Similar to OX40, 4-1BB is a co-stimulatory receptor expressed on T cells and antigen-presenting cells, and 4-1BB agonists enhance the anti-tumor effector functions of cytotoxic T cells (54). In preclinical studies using an MC38 colon carcinoma model, 4-1BB agonist therapy had little impact on tumor progression when administered early (less than 48 h after tumor implantation), while the combination of 4-1BB and anti-CTLA-4 together was effective even in established tumors (14 days after implantation) (55). However, this approach failed in B16 melanoma tumors (55), suggesting that the unique tumor microenvironment, in addition to tumor size, dictates therapeutic efficacy.

### Cancer Vaccines and Oncolytic Viruses

In contrast to ICB, where T cells are indiscriminately “rescued” from exhaustion regardless of antigen specificity, cancer vaccines aim to induce a tumor-specific adaptive immune response through delivery of whole tumor cells or tumor-derived antigens (56). Preclinical mouse models reveal cancer vaccine platforms are efficacious in small, but not large, tumors (21, 57). Coupling a bacteria Type II secretion protein with the model antigen ovalbumin (OVA), Binder and colleagues show that tumor rejection cannot be fully rescued in late stage B16 melanoma tumors, even with a strong OVA-specific CD8 T cell response (57). Similarly, two reports using killed tumor cells to vaccinate tumor-bearing mice find that late stage ovarian cancer or mammary tumors cannot be controlled, even with the addition of costimulatory or ICB mAbs (21, 58). These reports highlight the existence of a threshold of tumor burden below which the tumors are sensitive to immunotherapy, but above which the tumors are highly resistant.

Oncolytic vaccines offer the ability to prime T cell responses against tumor antigens *via* robust stimulation of the innate immune system. However, an adenovirus vaccine encoding tumor-specific somatic mutations (Gad-CT26-31) that works prophylactically or as a very early therapeutic intervention for small CT26 colon carcinomas was insufficient to eradicate large (>70mm^3^) established tumors (59). Similarly, a HPV vaccine resulted in the cures of small herpes poliovirus (HPV)-expressing TC-1 tumors whereas large tumors were more resistant, failing to achieve complete clearance (60). The inverse relationship between the success of viral vaccines or oncolytic viruses and high tumor burden holds true for patients with melanoma: treatment with the oncolytic virus talimogene laherparepvec, or T-VEC (which drives tumor cell killing and *in situ* priming against tumor antigens *via* cytokine release), results in better outcomes if tumor burden is low at the start of therapy (61). However, rational pairing of oncolytic viruses with ICB may hold promise: PD-1 blockade rescued anti-tumor immunity in mice bearing large tumors in both colon cancer and HPV models (59, 60). The potential beneficial impact of oncolytic viruses for diseases such as glioblastoma highlight the need to better understand the TME, to optimally pair treatment modalities for improved outcomes.

### Direct Antigen-Presenting Cell Activation

**Figure 1:**
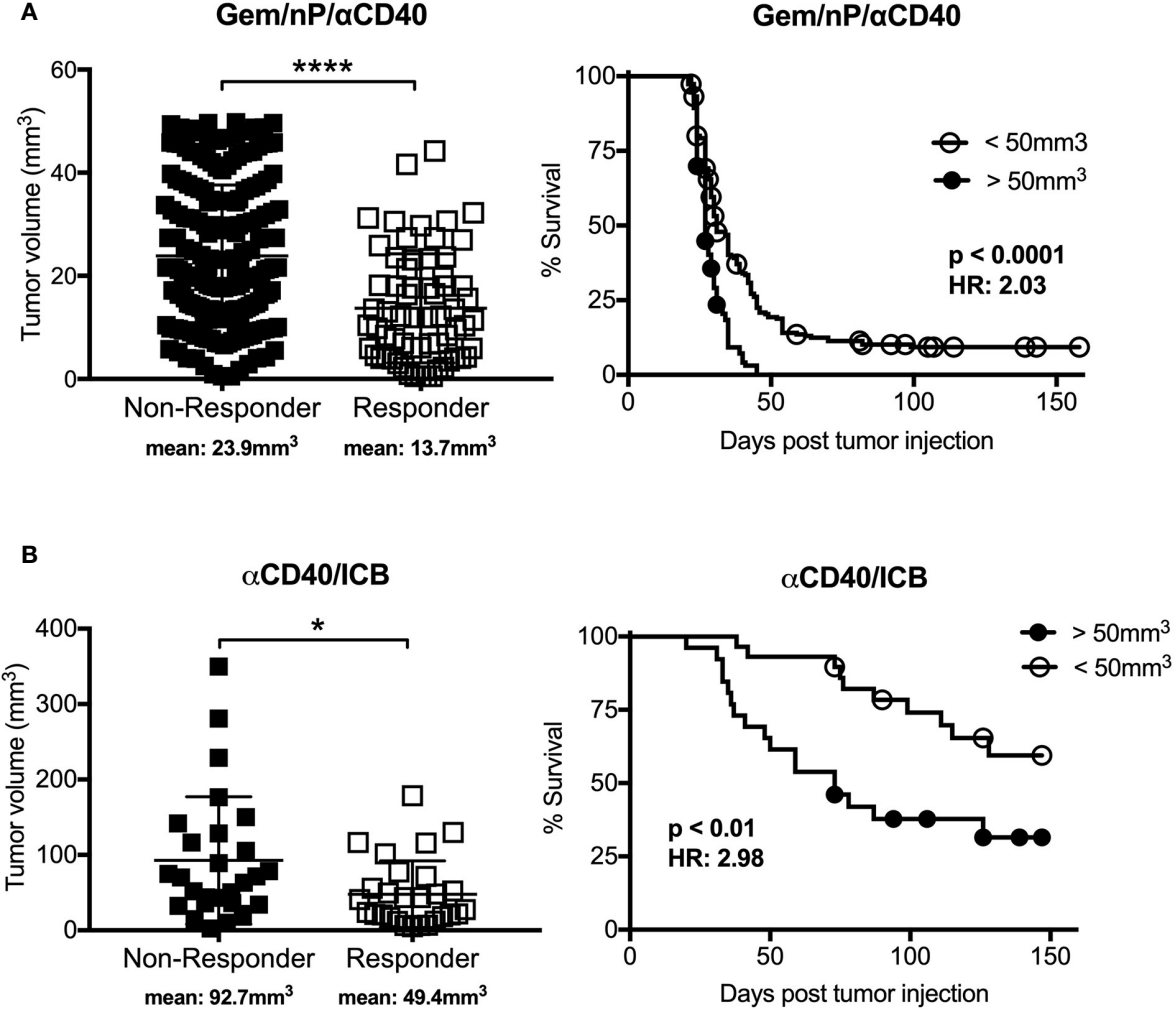
Efficacy of tumor rejection after combination treatment with agonistic CD40 antibody correlates with tumor size. Graphs from studies reported in (66) **(A)** and (67) **(B)**, reported here stratified by tumor response (defined as cured of primary tumor; scatter plots) or baseline tumor size (survival curves). Briefly, C57Bl/6 mice were implanted subcutaneously with 2.5x10^5^ 4,662 pancreatic ductal adenocarcinoma (PDAC) cell lines and treated with **(A)** agonistic CD40 (αCD40) in combination with chemotherapy (gemcitabine, Gem; and nab-paclitaxel, nP) as described in (66), or treated with **(B)** αCD40 in combination with αPD-1 and αCTLA-4 (ICB) as reported in (67). Each symbol represents a single mouse, horizontal lines indicate mean (also denoted below graph), and error bars indicate standard deviation. **** indicates p value < 0.0001 and * p < 0.05 as determined by an unpaired t test. For survival curves analysis was performed using log-rank test with indicated p values, HR indicates Hazard Ratio according to the size cutoff of 50mm^3^, calculated using log-rank. Analyses were performed using Graphpad Prism.

Cancer vaccines are often utilizing *ex vivo* DC activation, or *in vivo* innate immune sensors for activation of antigen presenting cells (APCs) to improve T cell priming, with the underlying hypothesis that insufficient T cell priming is one reason for the failure of ICB or co-stimulation mAbs to “rescue” T cell responses in the TME (62). However, direct activation of APCs such as by using an agonist CD40 mAb is another option for driving newly primed T cell responses against tumors. Ligation of CD40, expressed on APCs, promotes the rapid licensing and maturation of dendritic cells and other APC subsets independently of CD4 T cell help (the natural source of CD40 ligand) (63–65) normally provided upstream of immune checkpoint expression. We have shown that CD40 stimulation drives T cell immunity in a genetically engineered mouse model of PDAC when used in combination with chemotherapy (66), ICB (67), or combinations of chemotherapy and radiotherapy with ICB (68, 69), and there are promising data from a clinical trial in patients with metastatic PDA receiving agonistic CD40 in combination with chemotherapy (70). To assess the impact of tumor size in response to agonistic CD40 mAb therapy, we segregated tumor-bearing mice from previous studies according to therapeutic response and assessed the baseline tumor size in each group (66, 67). As shown in **Figure 1**, mice that responded to anti-CD40 in combination with chemotherapy (gemcitabine, Gem; and nab-paclitaxel, nP) or dual ICB had smaller baseline tumors than mice that were resistant to treatment. Mice with smaller tumors also survived longer in both treatment cohorts, although treatment with CD40/ICB resulted in slightly larger tumors responding to therapy as compared to mice treated with chemotherapy and CD40, supporting findings in cancer vaccine studies where a multi-pronged approach is more effective. Thus, non-redundant methods of stimulation T cell priming—directly *via* costimulatory mAbs, or *via* activation of APCs *via* CD40 mAb—still reveal the baseline tumor size as a major determinant of response.

### Adoptive and CAR T Cell Therapies

Given the systemic inhibition of immune responses in the presence of large tumors, adoptive cell therapy (ACT) is an appealing therapeutic option that bypasses the requirement for *in vivo* T cell priming or reactivation. Tumor-specific T cells are enriched *via ex vivo* expansion and activation before reinjection into the patient, and ACT of tumor infiltrating lymphocytes (TILs) has shown promise in metastatic melanoma (71, 72). However, tumor burden presents an issue even with antigen specific T cell therapies, as shown in a mouse model of lymphoma where ACT is effective only against small tumors (27). In large tumors, the authors found functional impairment and rapid deletion of the transferred CD8+ T cells, which was reversed *via* pretreatment with chemotherapy to reduce tumor size (27). Similar observations were made in a mouse model of sarcoma, where small tumors regressed after ACT, but large tumors on the contralateral flank did not (73). Recent studies show that the impairment and deletion of ACTs observed in large (but not small) tumors is reflective of suppressive local TME, as T cell affinity and expression of the tumor specific antigen was similar in both tumor sizes (74).

In addition to TILs, genetically modified T cells can also be used for ACT, including CAR T cells, where success has been made in many hematological malignancies, but solid tumors remain a more difficult problem (37). Hematological cancers such as acute lymphoblastic leukemia have a direct correlation between total tumor burden and response to CD19 CAR T cell treatment (75). In a mouse model of hepatocellular carcinomas, CAR T cells targeting glypican 3 were sufficient to drive regressions of small tumors but had no effect on large tumors, which was reversed upon the addition of sorafenib as a result of increased IL-12 production from intratumoral macrophages (76). In contrast, CAR T cells targeting glypican 1 were sufficient to drive tumor regressions in both large and small murine colorectal tumors (77). Thus the local TME can impact treatment efficacy (and can be targeted with rational therapeutic combinations) but the systemic impact on the immune response as a result of bearing large tumors is a significant barrier to treatment outcomes.

## Strategies to Reduce Tumor-Burden Associated Impacts on Immunity

Given that large tumors are more resistant to immunotherapy and harbor greater populations of suppressive cells, targeting tumor burden-associated alterations in the tumor microenvironment may exert therapeutic effects. Here, we briefly cover two major categories of approaches that may improve immune responses against tumors: directly reducing tumor size and modulating the tumor immune microenvironment.

### Traditional Interventions

#### Surgery

Tumor resection is the oldest method of treating cancer, and one of the original methods of restoring defective tumor surveillance in mouse models (1). The use of tumor resection prior to administration of immunotherapy improves control of tumor growth in multiple mouse models (3, 22, 78). This “resetting” of the immune response has been linked to the alleviation of immune suppression by tumor-derived IL-1 and granulocyte colony-stimulating factor (G-CSF) and could be phenocopied by mAb blockade of either cytokine (3). However, the trauma of surgical resection and the ensuing wound-healing process can lead to the outgrowth of otherwise immune-controlled metastases (79–81), which can be reversed by neoadjuvant administration of immunotherapies in some settings (81, 82). For example, patients with metastatic renal cell cancer benefited when surgery was combined with IFNα-2b treatment, resulting in a three-month increase in median survival with combination treatment compared to IFN treatment alone (83). In addition to immunotherapies, anti-inflammatory medication also prevents surgery-induced outgrowth of metastases by reducing the proportion of suppressive tumor-associated macrophages (82). Thus, surgical resection with careful management of the immunosuppressive wound-healing process could be considered prior to adjuvant immunotherapy.

#### Chemotherapy

Cytotoxic chemotherapy is another viable strategy to reduce tumor size that has been employed in the clinic for decades. Moreover, some chemotherapy regimens drive immunogenic cell death of tumor cells, leading to release of tumor antigens that can be presented by APCs to prime cytotoxic T cells (84). In this way, chemotherapy can exert both a local anti-tumor effect as well as systemic positive pressure on the immune system; these two outcomes are not mutually exclusive but highly interdependent. For example, a pre-clinical study showed that administering a low dose of the chemotherapy cyclophosphamide (CTX) one day before ACT of tumor-specific CD8 T cells is effective in reducing tumor burden and thereby prevents deletion of the transferred CD8 T cells in a murine lymphoma model (27). In the clinic, combination chemo-immunotherapy regimens have shown increased efficacy over chemotherapy alone in patients with advanced NSCLC (44) and metastatic melanoma (85).

However, given the cytotoxic nature of chemotherapy on any proliferating cell including T cells (86, 87), the impact of combination chemo-immunotherapy has variable results. The use of CTX followed by αCTLA-4 resulted in significant tumor regressions in a mouse model of colon cancer, while reversing the order resulted in apoptosis of tumor-reactive T cells (88), and we and others have observed similar detrimental impacts on sequencing gemcitabine with αCD40 in a mouse model of PDAC (89, 90). Furthermore, we have reported that the addition of the chemotherapy to agonistic CD40 and dual ICB significantly reduced the long-term survival of tumor bearing mice compared to immunotherapy alone (67). These findings suggest that chemotherapy, while important to restrain tumor growth, may not synergize with some types of immunotherapy. Additional investigations are necessary to determine optimal sequencing strategies such that chemotherapy can be employed to shrink large tumors without negatively impacting a developing immune response.

#### Radiation Therapy

Radiotherapy delivers high-energy rays directly to tumor sites as a form of curative control locally at the tumor, and the resultant abscopal effect can be beneficial for immune responses. The administration of radiation treatment completely ablates the tumor and surrounding stroma, effectively debulking the tumors. The immune impact is therefore complex—immune suppression may be alleviated by the eradication of the local tumor, while increased tumor cell death enhances systemic anti-tumor T cell response by generating novel tumor antigens presented on major histocompatibility complex (MHC)-I (91). In preclinical and clinical studies, the addition of radiotherapy to immunotherapy treatments such as ICB (92–94) and/or αCD40 (68) significantly improved immune response and survival. Mechanistically, radiation can upregulate expression of neoantigens (94), diversify the T cell repertoire (92), and enhance antigen presentation and co-stimulation on dendritic cells (68). Thus, in contrast to the detrimental immune impact of chemotherapy, radiation may promote immune cell activation while reducing tumor burden as a rational therapy partner for many immunotherapies.

## Simulating a “Small” Immune Microenvironment

### Physical Barriers to Immune Infiltration

Some of the highest hurdles for immune cell infiltration of the TME are the physical barriers of entry to the tumor site. Large tumors have reduced vascularization which creates regions of hypoxia that are particularly prominent in large tumors (95, 96), but reduced vascularization also compromises the delivery of a number of different types of therapies into the TME itself (97). This is further compromised when the TME is highly fibrotic, as is the case with PDAC and reviewed elsewhere in greater detail (98). While several mechanisms to target the stroma, such as inhibition of fibroblast activation protein (99), inhibition of Hedgehog signaling (100), or the use of chemotherapeutic agents such as nab-paclitaxel (101–103) have been devised, immune therapies can also be modified to overcome this barrier. For example, Maute et al. engineered a PD-1 ectodomain capable of blocking PD-1:PD-L1 interactions that is an order of magnitude smaller than anti-PDL1, and able to more effectively penetrate the TME (104). As a result, the PD-1 ectodomain was more effective against both large (150 mm^3^) and small (50 mm^3^) mouse CT26 colon carcinomas as compared to anti-PD-L1, which was only effective against small tumors (104). Thus, designing drugs with a higher degree of tumor penetration is a feasible approach for improved delivery of some immunotherapies.

### Overcoming Tumor-Associated Suppressive Cells

As discussed in Part I, the proportions of suppressive cell populations such as MDSCs are increased in large and progressing tumors. Blocking MDSC recruitment to or activity in the TME renders the tumor sensitive to immunotherapy [reviewed in depth elsewhere (105)]. We and others have shown that MDSCs are rapidly recruited to the PDAC TME *via* multiple chemokine and cytokine axes including CXCR2 (8, 106, 107), GM-CSF (108, 109), and G-CSF (8). Perturbation of these pathways reduces MDSC recruitment to the tumor site (8, 107, 108), increases CD8 T cells in the TME (8, 107, 108), and converts the tumor to immunotherapy-sensitive (8). MDSC reduction can be achieved *via* depletion, using CD33-specific immunotoxin Gemtuzumab ozogamicin (110) or ADH-503, a small-molecule agonist for CD11b (111), while the immunosuppressive activity of MDSCs can be blunted using anti-TIGIT (112) or CD73 blockade (113).

Tumor-associated macrophages (TAMs) play a similarly suppressive role in the TME and can also be targeted using depletion strategies. Colony-stimulating factor-1 (CSF-1), a key regulator of macrophage differentiation, can be blocked *via* CSF-1R mAb or antagonists, resulting in a sensitization of the TME to immune cell killing (114). Skewing TAMs to an anti-tumor functional profile also reduces the immunosuppression in hosts bearing large tumors. For example, pharmacological inhibition of PI3K-γ, which is highly expressed on myeloid cells, shifted TAMs from a M2-like immunosuppressive phenotype to an inflammatory M1-like phenotype in the murine 4T1 mammary carcinoma and B16 melanoma models (115). Paradoxically, reducing TAMs can result in a compensatory increase of MDSCs (116, 117). However, this effect can be overcome by blocking both TAMs and gMDSCs, which augments the efficacy of PD-1 blockade in a mouse model of cholangiocarcinoma (116) and bolsters chemotherapy in a mouse model of PDAC (117). The suppressive myeloid and macrophages subsets in the TME are thus a major immune cell barrier that must be overcome to generate effective anti-tumor responses.

Suppression in the TME is also closely regulated by Tregs, including both natural Tregs and tumor-induced Tregs. Selective depletion of Tregs by αCD25 shows promise in preclinical tumor-bearing animal models [reviewed in (118)], but can have unintended consequences of deleting effector CD25^+^ CD4 T cells. While effector CD4 T cell depletion does not negatively impact all immunotherapies (119), there is a growing appreciation of the contributions of CD4 T cells in mediating anti-tumor responses (67, 120–122). Some data suggest that αCTLA-4 specifically depletes CTLA-4^+^ Tregs in mouse models (123), but not in patients (124). Similarly, targeting glucocorticoid-induced TNFR family related protein (GITR) *via* agonistic antibody has also been shown to deplete Tregs in mouse models (125), while agonistic CD40 administration results in a drastic reduction in Tregs in the murine PDAC TME (66). These findings support targeting Tregs as well as other suppressive cell populations in the large TME to render the ratio of effector cell to suppressive cell more in alignment with ratios found in small tumor sites.

### Stimulating Immune Responses

In contrast to removing suppression, many immunotherapies specifically aim to promote immune activation and, to a certain extent, inflammation. The stimulation of innate immune signaling pathways is the original immunotherapy approach, used by William Coley in the early 1900s. “Coley’s Toxins” included a mix of bacterial products that he applied to the tumor lesions of patients, and he observed striking tumor regressions (126). Over the next century, immunologists deconstructed the immune response, resulting in the identification of cell types, receptors, and ligands that can be utilized to mount immune reactions.

Toll-like receptors (TLRs) are canonical pattern recognition receptors, and ligation by toll-like agonists stimulate the innate immune sensing pathways. In the context of the cancer-immunity cycle, providing TLR agonists licenses APCs to enhance antigen presentation and production of inflammatory cytokines (127). TLR agonists have significant anti-tumor roles by driving polarization of TAMs toward an anti-tumor, pro-inflammatory M1 phenotype (128). This conversion of TAMs to an M1 phenotype renders the TME more permissive to downstream activation of CD8+ T cells and infiltration to the tumor site naturally or *via* orthogonal combinations (129). Furthermore, TLR2 agonists alleviate the immunosuppression mediated by Tregs by reprogramming the Tregs to effector Th17 cells (130).

Despite these positive changes in the TME after TLR agonist administration, there are some tumor cells that express—and are activated—by TLR ligation (127, 131), which promotes tumor growth and chemoresistance in TLR7/TLR8 overexpressing human pancreatic cancer cells (132) and production of immunosuppressive cytokines (TGF-β, vascular endothelial growth factor (VEGF), IL-8) and resistance to apoptosis in human lung cancer cells (133). Implementation of TLR agonists may therefore have a number of benefits to reducing tumor suppression, but the type of TLR stimulated and the downstream impact may be regulated by the immunobiology of a specific TME.

A second class of innate immune sensor stimulation bypasses the binding of TLRs and instead directly stimulates the cyclic GMP-AMP synthase (cGAS) - stimulator of IFN genes (STING) pathway, which is triggered in the presence of cytosolic DNA (134). Activation of the cGAS-STING pathway in cancer cells leads to secretion of pro-inflammatory molecules that attract immune cells and restrict tumorigenesis (135, 136). Furthermore, tumor-cell derived DNA is transferred to APCs, activating the cGAS-STING pathway and production of type I interferons, leading to improved CD8 T cell priming (137, 138). STING activation has also been shown to act on tumor endothelial cells, leading to potent production of type I IFNs (139), further enhancing the anti-tumor response. STING agonists therefore offer an effective method of increasing CD8 T cell infiltration in the TME of large tumors, and have the potential to synergize in combination with a number of treatment modalities including ICB (140), VEGF receptor 2 blockade (141), and chemotherapy (142).

The TLR and STING agonist pathways are dependent downstream on signaling *via* type I IFNs. In contrast, CD40 stimulation bypasses the use of innate immune sensors and Type I IFN signaling, thus presenting an alternate bridge between innate and adaptive immunity (66, 67). Agonistic CD40 can drive the priming of a robust anti-tumor T cell response dependent on IFN-γ and Batf3 expression (8, 66, 67), while CD40 ligation on TAMs drives a tumoricidal response that depletes the stroma in the TME (143). As such CD40 activation is an important mechanism of increasing effector T cell trafficking to immunologically cold tumors, a finding that can be extended to treatment of large tumors that harbor more immunosuppressive cells and fewer effector immune cells.

### Oncolytic Viruses

Oncolytic viruses (OVs), originally designed to specifically kill tumor cells, have more recently been utilized as potent activators of local and systemic immune responses. OVs drive tumor cell death, resulting in the release of inflammatory signals (cell stress danger-associated molecular patterns or DAMPS) and the liberation of tumor-associated antigens to promote anti-tumor immunity. Most recently, clinical trials in patients with brain tumors have shown promising results using OVs (144), such as PVSRIPO (a modified poliovirus OV) in patients with glioblastoma, where some patients have shown complete and durable remissions (145). Future investigations into combinatorial approaches may further enhance the outcomes of OVs in tumors and help delineate which patients with brain tumors may require additional resections vs. orthogonal treatment combinations.

The immunostimulatory impacts of OVs can also be modulated by “cytokine-arming,” whereby the virus also contains a cytokine payload that re-educates, recruits, or stimulates immune cells locally in the TME [reviewed in (146)]. One highly successful example of a cytokine-armed OV is T-VEC, which selectively targets tumor cells while also producing GM-CSF (147). Mechanistically, T-VEC modulates the TME by activating and attracting tumor-specific CD8 T cells (148) *via* tumor cell lysis (resulting in the liberation of tumor antigens and DAMPs) and the recruitment of DCs [reviewed in (149)]. T-VEC has shown remarkable efficacy in clinical trials for patients with advanced melanoma both as monotherapy (150) and in combination with anti-CTLA-4 (151, 152). OVs thus have the potential to specifically target tumor cells for destruction while delivering a payload that promotes immune activation, a multi-pronged approach that may be ideal for large tumors that are otherwise not amenable to traditional tumor debulking approaches.

### Tumor Cell Intrinsic Mechanisms of Immune Regulation

In addition to contributing to aberrant tumor cell growth, oncogenic signaling pathways have the potential to regulate immune responses within the TME, suggesting that precision medicine in combination with immunotherapy should be explored for improved outcomes. For example, the Wnt/β-catenin signaling in a mouse model of melanoma hindered T-cell infiltration and established an immune “cold” TME by downregulating the expression of the chemokine CCL4 (153). Targeting the gene encoding β-catenin *via* RNA interference synergizes with dual ICB in a mouse model of breast cancer mice and results in complete tumor regressions (154), and small molecule inhibitors of Wnt signaling are being explored for potential application in tumor-bearing hosts (155). Similarly, we found increased expression of MYC in immune “cold” tumors, resulting in increased CXCL1 production and MDSC recruitment concomitant with a lack of T cells in the TME (8). JQ1 is a small molecule that binds competitively to bromodomains and inhibits MYC gene transcription by displacing bromodomain 4 (BRD4) (156), and has the added benefit of reducing PD-L1 expression by tumor cells and APCs (157) while also directly promoting T cell persistence and effector functions in the TME (157, 158). Although JQ1 shows promise in driving tumor responses when used in combination with ACT and ICB (158, 159), it has also been shown to activate and promote tumor invasion and metastasis pathways in mouse models of prostate cancer (160), suggesting further investigation is necessary for optimal application in the clinical setting.

There has been significant effort to develop effective inhibitors against the mitogen-activated protein kinase (MAPK) signaling pathway, given that it is one of the most commonly mutated pathways across all types of cancer (161). RAS proteins are activated upstream of MAPK and direct the establishment of the immunosuppressive TME *via* GM-CSF production (108) and upregulation of PD-L1 (162), but have proved difficult to target [reviewed elsewhere in detail (163)]. Interestingly, using ACT of T cells specific for an MHC I epitope derived from mutated Kras controlled tumor growth in a patient with metastatic colorectal cancer (164) and is being further investigated preclinically (165) and *via* Kras-directed cancer vaccine trials (163). Recently, the clinical development of Sotorasib, an inhibitor to mutant G12C KRAS, has yielding promising results in a phase I clinical study performed on patients with advanced solid tumors harboring the KRAS p.G12C mutation (166). It has also been shown to increase T cell, dendritic cell, and macrophage infiltration into the tumor site of CT26 KRAS G12C colon carcinoma-bearing mice, either alone or in combination with anti-PD-1 therapy (167). However, it is likely that tumor burden will continue to be a major barrier to these approaches as discussed above, and thus targeting RAS *via* immune-mediated mechanisms may require combinatorial approaches for optimal outcomes.

Targeting proteins downstream of RAS in the MAPK pathway has been more successful. BRAFV600E is one of the most common mutations, and a class of RAF monomer inhibitors specifically target this mutation to block mutant BRAF signaling (161). Vemurafenib is one such small molecule and administration significantly enhanced the survival of patients with metastatic melanoma harboring the BRAFV600E mutation (168). Critically, vemurafenib directly alters immune-associated features of malignant cells, such that blocking mutant BRAF signaling results in the reduction of suppressive cytokine production and increased MHC expression by tumor cells (169). This alters the local TME, resulting in decreased recruitment and survival of suppressive MDSCs (170) and Tregs (20), with a concomitant increase in MHC expression of melanocyte differentiation antigens [known targets of the endogenous T cell response against melanoma (171)] and with some BRAFi, T cell infiltration in to the TME (172). Thus BRAF inhibition controls tumor progression while also altering the tumor site toward a “small” TME, suggesting combination with other immune interventions may be highly effective.

Similar to BRAFi, mitogen/extracellular signal regulated kinase (MEK) inhibition controls tumor progression, but has been shown to negatively impact T cell priming and proliferation *in vitro* and in lymph nodes (173). However, MEKi appears to have a negligible negative impact on the local immune response against the tumor when administered *in vivo* (173). Indeed, MEKi increases infiltration of antigen-specific CD8 T cells into CT26 colon carcinoma tumors and protects intratumoral T cells from TCR-driven exhaustive apoptosis (174). These findings support the rational combination of MEKi with ICB, and accordingly, MEKi augments PD-1 or PD-LI blockade in mouse models of colon carcinoma and melanoma (174–176).

Despite the positive immune impacts of inhibiting mutant BRAF or MEK, many patients still progress after a period of tumor control, leading to the combination of BRAF/MAPKi in the clinical setting (177). This combination rendered tumors more sensitive to ICB, and adding PD-1 blockade further potentiated BRAF/MEKi in a mouse model of melanoma (178), and has been translated to the clinic for patients with mutant BRAF (179). Given the potential negative impact of MEKi on T cell priming in tumor-draining lymph nodes, rational immunotherapy combinations are crucial – e.g., pairing with CD40 stimulation may not be ideal while ICB or certain types of ACT may work very well with MEKi and these early combination trials are very promising.

### Epigenomic Modifiers

Abnormal gene expression is a hallmark of cancer progression, and histone deacetylases (HDAC), which regulate chromatin remodeling, are aberrantly expressed in many cancer types. HDACi results in cytostatic or cytotoxic effects on tumor cells, but this approach has been more effective in hematological malignancies than in solid tumors when used as a monotherapy or combined with other drugs including chemotherapy, reviewed by Suraweera and colleagues (180). Despite this, HDACi does reverse some of the gene repression found in tumor cells, thereby increasing expression of MHC and costimulatory molecules for both T and NK cells on tumor cells in the TME (181). In addition to tumor cell intrinsic effects, HDACi can also have direct effects on lymphocytes in the TME, similar to MEKi. Here the data are somewhat contradictory, as HDACi has differential impacts on T cell subsets, alternatively promoting IFN-γ production (182) and survival (183), while also restraining proliferation and cytotoxic functions *in vitro* (184). Importantly, tumor-associated Tregs are sensitive to Class I HDACi, with reductions in frequencies and suppressive function in a dose-dependent manner in a mouse model of renal cell carcinoma or prostate cancer (185). The impact of HDACi on APC subsets in the TME remains less clear, with some TAM subsets becoming less suppressive, other subsets acquiring an alternatively activated phenotype and DCs losing licensing and activation markers (181). These studies support the use of HDACi to help remodel the TME and associated immune response, but preclinical modeling will be crucial to elucidate the impact of HDACi on the initiation and maintenance of tumor immunity.

### Autophagy Inhibition

Autophagy is a mechanism by which cancer cells (and other cell types) degrade intracellular organelles as a source of nutrients under both basal and stress-induced conditions (186). Two FDA-approved drugs developed to treat malaria – chloroquine (CQ) and hydroxychloroquine (HCQ) – have been used extensively in the clinical setting but are also potent autophagy inhibitors. Initial *in vitro* studies revealed a cytotoxic impact after blocking autophagy in cancer cells and the first clinical trial was run in glioblastoma where patient survival was significantly extended when CQ was combined with radiation and chemotherapy (187), and CQ or HCQ have now been studied in the context of a number of different tumor types with somewhat variable results (186). In a mouse model of pancreatic cancer, a recent study highlighted the autophagy-mediated downregulation of MHC expression by tumor cells, which was reversible upon autophagy inhibition (188). Combining CQ with dual ICB drove potent tumor regressions and responses (188), revealing autophagy as a determinant of tumor cell immunogenicity. While this alone helps skew the TME toward a “small” TME phenotype with increased T cell infiltration (188), the benefits of systemic autophagy inhibition are much broader (186). CD8 T cells display enhanced anti-tumor effector function (189), while DCs upregulate MHC I expression after autophagy inhibition. However, tumor-intrinsic autophagy may be required for chemotherapy-induced tumor cell death, which can be a potent immune stimulator (190), highlighting the need for preclinical investigations to identify rational therapeutic partners for autophagy inhibition and successful immune responses against cancer.

## Concluding Remarks

**Figure 2:**
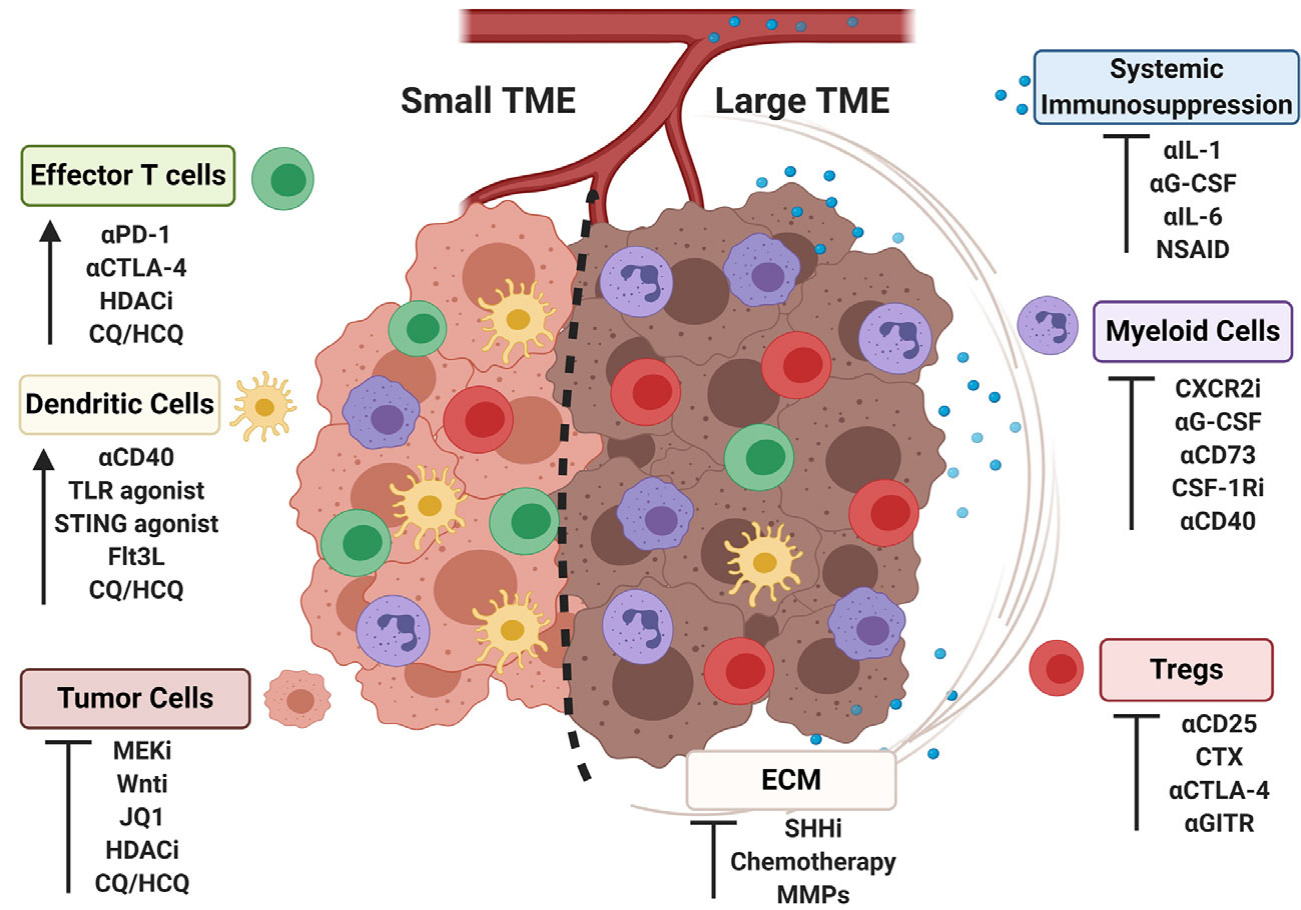
Strategies for skewing immune aspects of the large tumor microenvironment to a small tumor microenvironment. Representative cell populations frequencies found in large or small tumor microenvironments (TMEs) are shown (cell identities labeled on figure according to colors as shown). Strategies to target the TME are listed under cell populations known to response to specific interventions, with the outcomes indicated by an arrow or an inhibitory symbol.

Clinical and pre-clinical data indicate that tumor burden negatively correlates with response to a range of immunotherapies that include ICB, adoptive T cell therapy, and activation of antigen-presenting cells. Mechanistically, large tumors exert greater local and systemic changes to the immune system, and harbor more immunosuppressive cells and molecules that dampen antitumor activity. Many of the alterations locally and systemically reflect a more immunosuppressive tumor microenvironment; however, integrating this within the context of tumor burden may help stratify patients for optimally designed combination therapy approaches. Targeting features of the “large” TME can skew the immune microenvironment more toward phenotype of a “small” TME, rendering the tumor more sensitive to immunotherapeutic interventions as highlighted in **Figure 2**. A patient presenting with a large tumor burden that is amenable to debulking or size reduction to help alleviate system immunosuppression may require one approach, while a patient presenting with a relatively small but highly immunosuppressive tumor may require an entirely different therapeutic intervention. Small tumors that display an immunosupportive phenotype and are, at baseline, more amenable to immune interventions may require less invasive treatments than large tumors of the same type. Further studies interrogating the impact of bearing a large vs. small tumor are warranted, including investigations into the systemic impact on the immune response, the spatio-temporal localization of immune cell subsets within the TME as tumors progress, and opportunities to harness precision medicine with personalized immunotherapies. These investigations may reveal novel combinations of interventions that have the greatest impact on therapeutic outcome for patients with the greatest unmet clinical need.

## Author Contributions

SK and KB wrote the manuscript; all authors edited the manuscript. All authors contributed to the article and approved the submitted version.

## Funding

This work was supported by the Roy and Diana Vagelos Scholars Program in the Molecular Life Sciences at the University of Pennsylvania (SK) and Parker Institute for Cancer Immunotherapy (KB).

## Conflict of Interest

The authors declare that the research was conducted in the absence of any commercial or financial relationships that could be construed as a potential conflict of interest.
